# Facilitators and Barriers to Implementing a Remote Monitoring Model of Care for Stable Patients With Axial Spondyloarthritis Using the Consolidated Framework for Implementation Research: Qualitative Study

**DOI:** 10.2196/82480

**Published:** 2026-04-30

**Authors:** Yu Heng Kwan, Xin Ru Chew, Humairah Zainal, Zhonghui Xiong, Melissa Hock, Ka-Hao Zee, Ying-Ying Leung, Truls Østbye, Lian Leng Low, Hendra Goh, Charmaine Wai Yan Sum, Sungwon Yoon, Charmaine Tze May Wang, Warren Fong, Julian Thumboo

**Affiliations:** 1 Program in Health Services and Systems Research, Duke-NUS Medical School, 8 College Road, Singapore, 169857, Singapore, 65 90231226; 2 Department of Rheumatology and Immunology, Singapore General Hospital, Singapore, Singapore; 3 SingHealth Regional Health System, Centre of Population Health and Implementation Research, Singapore, Singapore; 4 Health Services Research Unit, Singapore General Hospital, Singapore, Singapore; 5 Lee Kong Chian School of Medicine, Nanyang Technological University, Singapore, Singapore; 6 Department of Family Medicine and Community Health, Duke University, Durham, NC, United States; 7 Post Acute and Continuing Care, Outram Community Hospital, Singapore, Singapore; 8 Yong Loo Lin School of Medicine, National University of Singapore, Singapore, Singapore

**Keywords:** axial spondyloarthritis, model of care, implementation science, consolidated framework for implementation research, patient reported outcome measures, Singapore

## Abstract

**Background:**

Close follow-up of stable patients with axial spondyloarthritis (axSpA) presents a financial burden and inconvenience to patients. A remote monitoring patient-reported outcome measures (PROMs)–based model of care (PROMise) was designed to reduce the frequency of in-person consultations for stable patients with axSpA. However, little is known about the facilitators and barriers of implementing a remote monitoring PROMise.

**Objective:**

This study aims to understand the facilitators and barriers, as well as the mitigation strategies to implementing a PROMise in the Singapore context.

**Methods:**

We conducted a qualitative study involving in-depth interviews with 19 patients with axSpA (78.9% (15) male, mean age 39.4, SD 11.7 years) and 13 health care professionals (HCPs) (23.1%, 3 male; mean age 37.9, SD 7.2 years) in a tertiary hospital in Singapore until data saturation was reached. Participants were purposively recruited based on sex, age, and ethnicity. Patients were additionally recruited based on the number of years since diagnosed with axSpA, while HCPs were recruited based on seniority and their role in the care of patients with axSpA. Interviews were transcribed, deductively analyzed, and mapped to the Consolidated Framework for Implementation Research (CFIR) framework to identify facilitators and barriers from both the patients’ and HCPs’ perspectives. The CFIR-Expert Recommendations for Implementing Change (ERIC) match tool was used to produce implementation strategies to overcome the CFIR barriers identified.

**Results:**

All five domains of the CFIR framework were elicited. Facilitators included (1) reduced inconvenience and costs for patients and reduced patient load in the clinic, (2) need for PROMise, (3) similarity to current workflows, and (4) suitable patient selection. Barriers included concerns for (1) financial sustainability of PROMise, (2) cultural conditions, (3) patient safety, and (4) increased workload for HCPs. In total, 35 ERIC strategies were matched to the corresponding CFIR barriers.

**Conclusions:**

We identified ERIC strategies that will facilitate the implementation of the PROMise model. In particular, focus should be placed on developing an implementation blueprint and obtaining continuous feedback from affected patients with axSpA and HCPs involved in the care of the affected patients. These implementation strategies cross-cut the CFIR barriers identified and thus may overcome the barriers to implementation.

## Introduction

Axial spondyloarthritis (axSpA) is a chronic inflammatory disease characterized by chronic back pain and stiffness of the pelvis and lower back. Peak disease onset occurs in late adolescence and early adulthood [1,2]. The reported prevalence of axSpA ranges from 0.3% to 1.4% [3,4]. Axial spondyloarthritis can have a long-lasting impact on various aspects of patients’ lives including in the functional, psychological, and social domains [5]. Hence, it is important to measure both the level of disease activity and the patient’s health-related quality of life (HRQoL), which can be achieved through patient-reported outcome measures (PROMs).

PROMs are standardized and validated questionnaires that provide patients’ perspectives of their health status and HRQoL over time. PROMs including the Bath Ankylosing Spondylitis Disease Activity Index (BASDAI) have indicated that a higher level of disease activity correlates with poorer HRQoL [6]. Patients with axSpA have a poor HRQoL that is comparable to that of patients with moderate to end-stage kidney disease [7]. It is important to diagnose axSpA early and closely follow up with patients with axSpA to prevent disease progression and improve HRQoL [8]. Furthermore, the use of disease‐modifying antirheumatic drugs such as sulfasalazine or biologics requires patients to be regularly monitored for toxicity and side effects. Hence, it is recommended that patients with axSpA visit a rheumatologist at least twice a year [9].

However, frequent visits to the rheumatologists result in both a high economic cost to patients and inconvenience due to time spent traveling and waiting for consultations [10]. Patients with axSpA spend on average US $180 per visit to the rheumatologist [11]. Hence, there is a need for an alternative monitoring strategy. A randomized controlled trial by Hermans et al [12] in the Netherlands remotely monitored patients with axSpA through laboratory tests and PROM results. In that study, patients were able to self-initiate appointments after a review of their laboratory test and PROM result by a rheumatologist. The model was shown to reduce health care costs and be noninferior to usual care in terms of quality-adjusted life years in the remote monitoring group.

**Figure 1.**
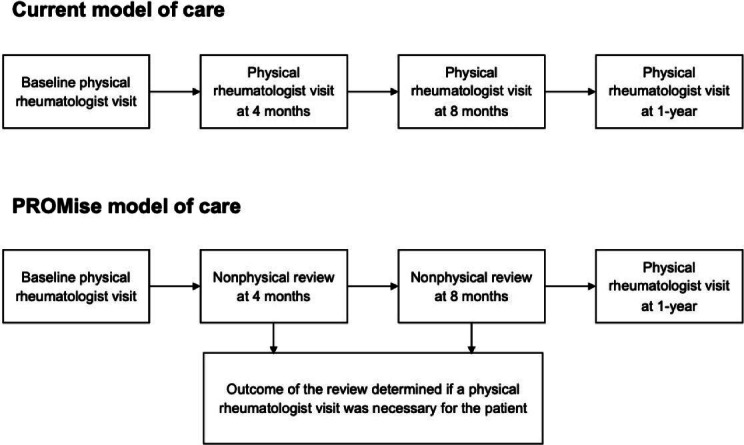
Comparison between the model of care and patient-reported outcome measures–based model of care (PROMise). AMS: automated monitoring system; PROMise: patient-reported outcome measures–based model of care.

We propose a similar PROMs-based model of care (PROMise), which aims to remotely monitor stable patients with axSpA through an automated review of their laboratory test and PROM results. Patients are not required to be physically or virtually present in the hospital for the automated review (see Figure 1). Through an automated review of the laboratory test and PROM result, we aim to maintain the standards of patient outcomes and patient experience while reducing health care costs and improving health care use [12].

However, the implementation of a new model of care is a complex and challenging process [13]. It has been frequently stated that it takes an average of 17 years for research evidence to reach clinical practice [14]. Hence, implementation science provides a unique opportunity to expedite the time from evidence generation to clinical implementation [15]. The Consolidated Framework for Implementation Research (CFIR) is a theoretical framework commonly used in implementation science. It consists of 5 major domains (innovation, outer setting, inner setting, individuals, and implementation process) [16]. The Expert Recommendations for Implementing Change (ERIC) is a compilation of 73 implementation strategies that can be used in isolation or in combination with CFIR to tailor implementation strategies [17]. The CFIR-ERIC matching tool links both key resources, allowing users to use ERIC strategies to overcome the identified CFIR barriers [18]. There was a qualitative study by Smits et al [19] in the Netherlands that evaluated the experiences of patients with axSpA and health care professionals (HCPs) with a remote monitoring model of care in axSpA. However, the study did not have an implementation science focus, and it is important to evaluate implementation processes in a local context to ensure innovations can be implemented successfully and sustainably [20].

Therefore, we aimed to understand the facilitators and barriers to implementing a PROMise model in the Singapore context through the CFIR framework and mitigate the CFIR barriers identified through implementation strategies matched by the CFIR-ERIC matching tool. The insights gained from this study will facilitate subsequent implementation of the PROMise model for stable patients with axSpA in Singapore.

## Methods

### Study Design and Setting

This was a qualitative study relating to the proposed implementation of the PROMise model. The study was conducted in the outpatient rheumatology clinic in Singapore General Hospital. Singapore General Hospital is the largest tertiary hospital in Singapore and serves patients from across the country. In-depth interviews were conducted from November 2024 to May 2025 to elicit both the HCPs’ and patients’ views on the potential implementation of the PROMise model.

The proposed PROMise model remotely monitors patients with axSpA through an automated review of laboratory tests and PROM results. Stable patients will be identified during their routine visits based on a BASDAI score of lower than 4, which indicates that disease activity is inactive in the patients [21]. The patients will then be given a PROM questionnaire, in this case BASDAI, to fill out prior to their upcoming automated review. The BASDAI questionnaire can be completed remotely in the patients’ homes. The schedule of their next appointment will then be determined algorithmically based on the outcome of their automated review (see Figure 1). Patients will have a minimum of one in-person visit to the rheumatologist per year. The PROMise model is under development at the time of this writing, and once refined, is expected to be integrated as part of routine assessment in all future follow-up visits.

### Participants

Patients were eligible if they were aged 21 years and older and had been diagnosed with axSpA by a rheumatologist. All patients fulfilled the 2009 Assessment of SpondyloArthritis International Society criteria with a BASDAI score of <4 [2]. HCPs were eligible if they were caring for a patient with axSpA at the time of this writing. HCPs had a diverse variety of roles in the routine care of patients with axSpA; those that could potentially be affected by the implementation of the PROMise model were of particular interest. This included rheumatologists, nurses, allied health professionals, and clinic operations staff. Participants were purposively sampled from a list of patients, who were screened based on eligibility criteria. Purposive sampling was used to ensure representation across key demographic and clinical characteristics. Both patients and HCPs were purposively recruited based on sex, age, and ethnicity. Patients were additionally recruited based on the number of years since diagnosed with axSpA, while HCPs were recruited based on seniority and their role in the care of patients with axSpA. A sampling matrix that included gender, age, ethnicity, and years since diagnosis for patients and years of clinical experience and professional role for HCPs was used to monitor key participant characteristics throughout recruitment. Recruitment was iteratively adjusted to ensure diversity across these characteristics, with targeted efforts made to include underrepresented groups. The eligible patients were approached in person at the outpatient rheumatology clinic by a research staff who explained the purpose of the study.

### Data Collection

The CFIR framework was used as a guide to design a standardized, semistructured interview guide (Multimedia Appendix 1) [22]. Individual in-depth interviews were conducted and audio-recorded for both HCPs and patients by 2 trained facilitators (HZ and XRC). Field notes were taken when needed. The interviewers were not part of the care team for the participants. The interviews lasted around 30 minutes on average and were conducted remotely via online video conferencing. Data saturation was defined as the point at which no new codes or subthemes emerged within the CFIR domains represented in this study. Saturation was achieved with at least one code or subtheme identified per domain and confirmed through 3 additional interviews for both patients and HCPs, which did not yield new codes or subthemes within these domains. We also collected basic demographics such as age, sex, education, and ethnicity, as well as the number of years since axSpA was first diagnosed for patients and the number of years in their position at the time of this writing for HCPs.

### Data Analysis

The interviews were transcribed verbatim, and the transcripts were checked against the initial audio recording by the coders (HZ, MH, ZX, and XRC) to ensure accuracy and enhance familiarity with the dataset. Coding was performed by independent coders who worked in pairs. Transcripts were thematically analyzed based on Braun and Clarke [23-25], adopting a structured, codebook-based approach and ensuring clarity and transparency in analytic procedures. Each coder independently reviewed transcripts and generated initial codes. The coders then met to compare coding, discuss differences, and organize codes into potential categories and subthemes. A codebook was developed and iteratively refined to document coding definitions, decisions, and subtheme development. Coding discrepancies were discussed and resolved through consensus, with input from a third researcher (YHK) where required. Regular team discussions were held to review and refine the coding framework and thematic structure to enhance analytic rigor. To enhance trustworthiness, several strategies were used. Credibility was supported through investigator triangulation and repeated engagement with the data. Dependability was strengthened by maintaining an audit trail of coding decisions and codebook development. Confirmability was addressed through team-based discussions to minimize individual bias. Transferability was facilitated by providing contextual details of the study setting and participants to enable assessment of applicability to other contexts. NVivo software (version 15; Lumivero) was used for data management. The reporting of qualitative methods followed the Consolidated Criteria for Reporting Qualitative Research (COREQ) checklist (Checklist 1) [26].

The CFIR-ERIC matching tool was used to develop and outline the expert-endorsed Level 1 and 2 strategies. The identified CFIR barriers were input into the CFIR–ERIC matching tool. The tool’s algorithm is derived from an expert consensus panel that ranked how well different implementation strategies address specific CFIR barriers. It then generates a prioritized list of strategies by aggregating the importance ratings assigned by experts across the selected domains [17]. In the development of the CFIR-ERIC matching tool, experts were invited to endorse their top 7 strategies for overcoming a specific CFIR barrier. A total of 18 Level 1 and Level 2 strategies were endorsed by more than 50% of experts and between 20% and 50% of experts, respectively. The CFIR-ERIC output was then analyzed to determine suitable implementation strategies to overcome the main barriers identified.

### Ethical Considerations

This study was approved by the SingHealth Centralized Institutional Review Board (Ref No. 2024‐4044). Informed written consent was obtained from all participants before the interviews. All data collected from participants were deidentified. As a token of appreciation, each participant received an SGD $50 (US $39.34) coupon upon completion of the interview. The study was conducted in compliance with the principles of the 1964 Declaration of Helsinki.

## Results

### Participant Characteristics

A total of 32 interviews were conducted. The participant characteristics are summarized in Table 1. The mean (SD) age for patients was 39.4 (11.7) years and for HCPs was 37.9 (7.2) years, respectively. The HCPs were employed in a diverse range of roles in the clinical setting, from clinic operation staff to rheumatologists; all were involved in the care of patients with axSpA. The HCPs had between 1 year and 23 years of service (Table 1).

**Table 1.** Participants characteristics.

Participants		Values
Patients (n=19)		
Age (years), mean (SD)		39.4 (11.7)
Sex, n (%)		
Female		4 (21.1)
Male		15 (78.9)
Race, n (%)		
Chinese		13 (68.4)
Non-Chinese		6 (31.6)
Education, n (%)		
Secondary		2 (10.5)
Tertiary		17 (89.5)
Housing, n (%)		
Private		6 (31.6)
Public		13 (68.4)
Marital status, n (%)		
Married		9 (47.4)
Single		10 (52.6)
Occupational status, n (%)		
Employed		16 (84.2)
National Service		1 (5.3)
Student		1 (5.3)
Unemployed		1 (5.3)
Number of years since diagnosed with axSpA^a^, mean (SD)		8.05 (5.21)
HCPs^b^ (n=13)		
Age (years), mean (SD)		37.9 (7.2)
Sex, n (%)		
Female		10 (76.9)
Male		3 (23.1)
Race, n (%)		
Chinese		9 (69.2)
Non-Chinese		4 (30.8)
Education, n (%)		
Tertiary		13 (100.0)
Housing, n (%)		
Private		5 (38.5)
Public		8 (61.5)
Marital status, n (%)		
Married		10 (76.9)
Single		3 (23.1)
Occupational status, n (%)		
Employed		13 (100.0)
Professional role, n (%)		
Clinic operations		3 (23.1)
Doctor		6 (46.2)
Nurse		2 (15.4)
Pharmacist		2 (15.4)
Number of years in the role, mean (SD)		7.7 (6.6)

a axSpA: axial spondyloarthritis.

b HCP: health care professional,

### Facilitators Mapped to CFIR Constructs

Using CFIR as the a priori framework for identifying the barriers and facilitators to implementation of the novel PROMise, all 5 domains (innovation, outer setting, inner setting, individuals, and implementation process) were elicited. We identified 4 main facilitators to the implementation of the PROMise model, which are presented in Table 2.

**Table 2.** Facilitators of implementing a patient-reported outcome measures (PROMs)–based model of care mapped to the Consolidated Framework for Implementation Research (CFIR) framework.

CFIR domain	Perceived facilitator	Representative quotes
Innovation (relative advantage)	Reduced inconvenience and cost Reduced patient load	“What I’m looking forward to is the time that I can save … otherwise it’s just like half to full day gone … also maybe a little on the finances’ side because sometimes I would have to take the taxi down to the hospital.” (Patient, Female, 36) “So I mean most of us are very busy, so it would save me the time to go down, yeah. And of course, there will be cost savings right, because we don’t incur the cost of going down there.” (Patient, Male, 46) “The patients’ laboratory tests are still being taken, so it is a win-win. The patient feels that someone is looking out for their symptoms and test results. At the same time, I’m spending less clinic time attending to them while they still are feeling okay and satisfied with the care.” (HCP^a^, Female, 42) “… reduce the load for clinic with consultations … but they are able to detect any symptoms changes and allow for timely intervention. I would say it reduces everybody’s workload, and still effectively allows patients to access hospital’s treatment if needed.” (HCP, Female, 33)
Inner setting (tension for change)	Need for the new model of care (PROMise)^b^	“The clinic slots are very limited. So that’s why we are so overwhelmed in clinic itself, because every almost doctor needs to triple book, double book appointments. … at least release some stress from the workload and then the more critical care patients can be seen on time.” (HCP, Female, 44) “The schedule sometimes hinders me, because I need to adjust to the doctor’s schedule … because currently I’m working on Mondays to Fridays. … and there’s no clinic during Saturday and Sunday.” (Patient, Male, 24)
Inner setting (compatibility)	Similarity to the workflows	“The process itself is fine, it’s already like the old scenario of me coming down for the physical appointment, doing the physical PROMs^c^ sheet, and then seeing the doctor’s name and doing the blood test as part of the process.” (Patient, Male, 30)
Individuals (innovation recipients)	Suitable patient pool selection	“I think it is good that we are starting out with the SpA^d^ patients because they are relatively young … and their conditions can also be relatively new where they do not have many complications and comorbidities.” (HCP, Female, 34) “… patients with axial spondyloarthritis are also sort of a special group because they tend to be younger, so they are more informed and they can better monitor themselves, so they know when things are not too good and they want to come in earlier, so that’s good.” (HCP, Male, 45)

a HCP: health care professional.

b PROMise: patient-reported outcome measures–based model of care.

c PROM: patient-reported outcome measure,

d SpA: spondyloarthritis.

#### Innovation (Relative Advantage)

Many patients believed that they would be able to save on the costs and time spent traveling to the hospital. In particular, some patients *“*look forward to … the time that I can save … otherwise it’s just like half to full day gone.” (Table 2). Hence, they felt that the remote monitoring model of PROMise would be better than their schedule of physical appointments at the time of this writing.

In addition, HCPs felt that the PROMise model would be able to reduce the patient load in the clinic. Many HCPs also pointed out that even though they will be “spending less clinic time attending” to patients, the patients will still be closely monitored (Table 3). HCPs believe that PROMise will still be “able to detect any symptom changes and allow for timely intervention” (Table 2).

**Table 3.** Barriers of implementing a patient-reported outcome measures (PROMs)–based model of care mapped to the Consolidated Framework for Implementation Research (CFIR) framework.

CFIR domain	Perceived barrier	Representative quotes
Innovation (cost)	Financial sustainability concerns	“… if this is an incentive for the patient does it mean that it is a disincentive for the organization, … I’m not sure in the future how we will charge our patients for PROM^a^ based care. Because manpower is still being pumped to review these blood test results, PROMS results, …” (HCP^b^, Female, 34) “My only question would be how much it would cost, and whether that would be a barrier to us talking to the senior management.” (HCP, Female, 41)
Outer setting (local conditions)	Cultural concerns	“I think the elderly will be a bit more reluctant to take part in this … because of the language and their eyesight, it may be a bit difficult for them and they are not well versed in the technology …” (Patient, Male, 62) “… language barrier regarding the questionnaires? … if the patient only speaks like dialect or non-English, non-Mandarin then someone has to interpret for them, I think that would be difficult…” (HCP, Female, 44) “… I hope they are not deprived of the opportunity to take their time to transition or not transition at all. So, I just hope there’s a transition period if there is going to be a change of in the model of care.” (Patient, Male, 30)
Individuals (needs)	Patient safety concerns	“… but I think if there’s a urgent medical condition, maybe it should schedule the appointment earlier ... I think the patients should be informed straight away to go for an earlier appointment?” (Patient, Male, 62) “.. have a direct helpline to contact the medical staff, … immediately contact the nurse … and they can quickly review or share your results with the doctor and get that assurance. I think if that’s done well, it wouldn’t compromise my safety or well-being.” (Patient, Male, 30)
Implementation process (planning)	Increased workload concerns	“… for the patients to be seen in the SpA^c^ Clinic within two weeks, that’s a little too short. I don’t think that the SpA patient needs to be seen so urgently, .... So perhaps four to eight weeks would be more reasonable ... Because the clinician also have very busy clinics that they cannot at two weeks’ notice, slot someone in...” (HCP, Female, 42) “What happens if that they don’t do the blood test or they don’t answer the questionnaire, then what happens to them? So the workflow process has to be there, and if there’s administrative support for this, then that will be good… if the clinician is to be part of this workflow process, then time has to be set aside to vet through all the results of the patients.” (HCP, Male, 45)

a PROM: patient-reported outcome measure,

b HCP: health care professional.

c SpA: spondyloarthritis.

#### Inner Setting (Tension for Change)

Both patients and HCPs felt that there was a need for change in the current model of care. HCPs felt that they were overburdened with clinical load and thus had to “triple book, double book appointment” slots to be able to accommodate the patient numbers (Table 3). Additionally, patients were also unsatisfied with the inconvenience of attending physical consultations. A few patients mentioned that they often face scheduling conflicts when attending physical appointments as they have to “adjust to the doctor’s schedule” (Table 2).

#### Inner Setting (Compatibility)

Patients pointed out that the necessary tasks of “doing the blood tests” and “doing the physical PROMs sheet” were already present in this model of care (Table 3). At the time of this writing, all patients are required to complete laboratory tests and the BASDAI questionnaire before their physical consultation. Many patients pointed out the differences would be that first, the questionnaire will now be administered digitally, and second, a physical appointment may not be required depending on the outcome of the automated review.

#### Individuals (Innovation Recipients)

HCPs mentioned that patients with axSpA were well suited for the PROMise model. Patients with axSpA were *“*relatively younger” and hence “do not have many complications and comorbidities” that would require closer monitoring from HCPs (Table 3). Furthermore, it has been raised that the patients with axSpA can *“*better monitor themselves, so they know when things are not too good and they want to come in earlier” and are able to seek the necessary medical attention if their condition worsens (Table 2).

### Barriers Mapped to CFIR Constructs and Level 1 ERIC Strategies

We identified 4 main barriers to the implementation of the PROMise model (Table 3).

#### Innovation (Cost)

Some HCPs reported that they were concerned with the financial sustainability of the PROMise model. They pointed out that resources such as *“*manpower is still being pumped*”* to review the laboratory test and PROMs results; however, they were unsure if these costs were chargeable to the health care system (Table 2). HCPs were worried that the cost of the PROMise model will be unsustainable in the long term as the sources of funding for the PROMise model are still unclear. The lack of clarity regarding the financial sustainability of PROMise will be *“*a barrier to [the HCPs] talking to the senior management” to seek approval for the PROMise model (Table 2).

The Level 1 ERIC strategy recommended to mitigate the barrier of financial sustainability concerns is to access new funding (82%; Table 4).

**Table 4.** Level 1 and 2 strategies identified by the Consolidated Framework for Implementation Research-Expert Recommendations for Implementing Change (CFIR-ERIC) matching tool.

ERIC^a^ strategies^b^	Cumulative percent^c^	Cost	Needs	Planning
**Conduct local needs assessment**	**111%**	4%	**57%**	**50%**
**Assess for readiness and identify barriers and facilitators**	**92%**	16%	33%	42%
**Develop a formal implementation blueprint**	**86%**	8%	5%	**73%**
**Obtain and use patients or consumers and family feedback**	**84%**	4%	**76%**	4%
**Access new funding**	**80%**	**72%**	—^d^	8%
**Involve patients and family members**	**75%**	—	**71%**	4%
**Alter incentive or allowance structures**	**65%**	44%	10%	12%
**Conduct local consensus discussions**	**56%**	4%	29%	23%
Prepare patients to be active participants	48%	—	48%	
Identify and prepare champions	48%	12%	5%	31%
Develop and implement tools for quality monitoring	45%	—	14%	31%
Use advisory boards and workgroups	44%	—	29%	15%
Tailor strategies	38%	12%	14%	12%
Develop resource sharing agreements	36%	32%	—	4%
Make billing easier	32%	32%	—	
Intervene with patients to enhance uptake & adherence	32%	4%	24%	4%
Facilitation	31%	8%	—	23%
Promote adaptability	30%	16%	14%	—
Conduct educational meetings	29%	12%	10%	8%
Conduct cyclical small tests of change	29%	8%	10%	12%
Capture and share local knowledge	29%	4%	10%	15%
Fund and contract for clinical innovation	28%	28%	—	—
Involve executive boards	25%	20%	5%	—
Provide local technical assistance	24%	4%	5%	15%
Use an implementation adviser	24%	4%	5%	15%
Place innovation on fee for service lists or formularies	24%	24%	—	—
Model and simulate change	24%	20%	—	4%
Visit other sites	24%	16%	—	8%
Stage implementation scale up	23%	8%	—	15%
Conduct ongoing training	23%	—	—	23%
Build a coalition	22%	4%	14%	4%
Increase demand	22%	12%	10%	
Purposely reexamine the implementation	20%	—	5%	15%
Alter patient fees	20%	20%	—	—
Use other payment schemes	20%	20%	—	—
Identify early adopters	20%	8%	—	12%

a ERIC: Expert Recommendations for Implementing Change.

b Bolded strategies indicate Level 1 strategies, defined as those endorsed by a majority of experts (>50% agreement), whereas unbolded strategies indicate moderate agreement (20%–50%).

c Bolded values indicate that a majority of experts (>50% agreement) endorsed the strategy as a mitigation approach for the corresponding CFIR construct, whereas unbolded values indicate moderate agreement (20%–50%).

d Not applicable.

#### Outer Setting (Local Conditions)

Both patients and HCPs had concerns that some patients would experience challenges with the digital platform, particularly for older adults. Technological proficiency varies across ages, and there is a common perception that the older adult patients “are not well versed in the technology” (Table 3). Hence, they will not be able to readily access the digital PROMs questionnaire.

Both patients and HCPs have also shared concerns regarding language literacy. Patients who do not understand English will not be able to answer the English-based PROMs questionnaire. They would then require *“*someone to interpret [the questionnaire] for them,” which would be an additional hurdle to the implementation of PROMise (Table 3).

One patient was concerned that other patients may not be able to adapt to the new model of care due to similar concerns relating to digital and language literacy mentioned above. This patient hoped that such individuals would be given an “opportunity to take their time to transition or not transition at all” and that there should be a *“*transition period” to ease patients into the new model of care (Table 3).

#### Individuals (Needs)

A few patients were concerned that the model of care will not meet their needs, and they will not receive timely medical attention. They were worried that flares or symptoms that worsen or newly emerge may not get the necessary medical attention in time.

Patients suggested establishing “a direct helpline” to the clinic that will allow for timely access to medical assistance, indicating that it would alleviate their safety concerns (Table 3).

The Level 1 ERIC strategies recommended to mitigate the barrier of concerns for patient safety are to involve patients and families (71%), use patients’ and families’ feedback (76%), and conduct a local needs assessment (57%; Table 4).

#### Implementation Process (Planning)

Some HCPs raised concerns that their workload would increase under this new model of care. As dedicated “time has to be set aside to vet through*”* the laboratory data and PROM results from the patients, in addition to the workload that the HCPs have (Table 3). HCPs also worry that they will be unable to accommodate the sudden influx of patients requiring follow-up due to their overbooked clinics.

The Level 1 ERIC strategies recommended to mitigate the barrier of additional workload concerns are to develop a formal implementation blueprint (73%) and conduct a local needs assessment (50%; Table 4).

### Strategies Generated by CFIR-ERIC Matching Tool

Overall, the CFIR-ERIC matching tool produced a total of 35 Level 1 and Level 2 strategies (Table 4). There were 8 Level 1 strategies, which had expert agreement of more than 50%. Of the 8 Level 1 ERIC strategies, 7 strategies cut across all 3 barriers of innovation cost, individuals (need), and planning. The strategy of accessing new funding cut across only the barriers of innovation costs and planning, as it did not address the needs of patients. The patients required additional reassurance that they would still receive timely medical attention under this model of care, which could not be resolved by accessing new funding.

The cumulative percentages exceeded 100% because experts were invited to endorse the same implementation strategy for multiple CFIR barriers independently.

## Discussion

### Principal Findings

This study sought to identify and understand the facilitators and barriers to implementing a novel model of care for patients with axSpA (PROMise) from both the perspectives of patients and HCPs using the CFIR framework. Facilitators included (1) reduced inconvenience and costs for patients and reduced patient load in the clinic, (2) need for an alternative model of care, (3) similarity to these workflows, and (4) suitable patient selection for PROMise. Barriers included concerns for the (1) financial sustainability of PROMise, (2) cultural conditions, (3) patient safety, and (4) increased workload for HCPs. To the best of our knowledge, this is the first study to break down the facilitators and barriers to the implementation of a novel model of care and suggest implementation strategies to address barriers identified prior to the formal implementation of the novel model of care. This study provides insights for future studies that aim to implement remote monitoring strategies in axSpA or other chronic diseases.

The findings of cost and increased workload being barriers are common in the implementation process [27,28]. In our study, even though there were concerns with the direct costs of the new model of care, the greater concern of the HCPs was the lack of clarity regarding the financial structure of the proposed model of care. HCPs also mentioned a lack of clarity on how to set aside dedicated time to review patients’ laboratory tests and PROMs results who require follow-up in the PROMise model. Although both ERIC strategies of accessing new funding and developing an implementation blueprint were recommended for the mitigation of the abovementioned barriers, developing an implementation blueprint would be more relevant in this particular context. The key features of the implementation blueprint should include cost evaluation, patient billing, and detailed workflow for not just clinicians but all HCPs involved in the PROMise model. Hence, an implementation blueprint would shed light on the financial sustainability of the model of care and increase workload transparency for the HCPs [29].

Both patients and HCPs brought up considerations regarding technological and language literacy. Poor technological literacy has been described in local publications [30]. Even though the vast majority of Singapore residents are literate in English (97.1%), there is still a small minority who will face challenges completing the PROM questionnaire in English [31]. For patients who either have challenges in technological or language literacy, more efforts will be needed to determine how support can be provided. Furthermore, these patients are likely to be older and have an increased likelihood of co-morbidities, hence requiring closer monitoring from HCPs [32,33]. However, patients with axSpA are likely to be younger, as pointed out by the HCPs. Hence, the majority of the patients with axSpA will still benefit from the PROMise model. For the small minority of patients with lower technological and language literacy and/or more severe illness, it is likely a longer transition period will be required to ease these patients into the PROMise model as suggested, or to determine if they are suitable at all.

Patient safety was a concern for a minority of patients. Patients desired a direct avenue to seek help in between the scheduled reviews by the algorithm. This was in contrast to findings by other studies where other patients felt a sense of security under a remote monitoring model of care [34]. However, patients in this study did mention that their concerns would be greatly alleviated if they were able to contact the clinic staff directly regarding the scheduling of appointments if they experience worsening symptoms. Hence, the future implementation of the PROMise model should include and inform patients of the access to a direct helpline to address their safety concerns.

With regard to facilitators from the patients’ perspectives, the benefits of remote monitoring are well documented. Our study found similar facilitators such as patient convenience and reduced traveling costs [35,36].

Based on the recommended ERIC strategies of conducting a local needs assessment, assessing readiness, and identifying barriers and facilitators; and involving, obtaining, and using patient and family feedback, continuous feedback from patients and HCPs will be collected and used to improve the PROMise model. The continued feedback from patients and HCPs is integral to the sustainability of the PROMise model after implementation [37].

### Strengths and Limitations

The strengths of this study include purposive sampling of the HCPs involved in the care of patients with axSpA in Singapore General Hospital. We ensured the inclusion of a wide range of ages, levels of clinical experience, and diverse clinical and nonclinical roles that would be affected by the implementation of the PROMise model. Therefore, our data captured a broad range of views and was representative of the views of HCPs that would be affected by this model of care.

There are a few limitations to our study. First, our study focused on interviews with patients and HCPs involved in the care of patients with axSpA. We lack representation of other stakeholders such as the health care leadership. These stakeholders may have provided a more comprehensive understanding of the barriers and facilitators of the implementation process. However, we tried to mitigate this by including staff involved in operations to understand the barriers and facilitators to the implementation of PROMs. Moving forward, we will solicit feedback during our meetings with the health care leadership. Second, as the proposed PROMise model of care has not been piloted yet, the patients and HCPs interviewed have not had first-hand experience with the model of care. Hence, the perspectives that they provided were only based on their perception of what a PROMise is. They may have a different perspective after they have experienced the model of care personally, especially after the implementation process has been tailored according to recommendations. Hence, we will consistently solicit feedback from patients and HCPs after the model of care has been piloted to better understand and improve upon the implementation process.

### Conclusion

Our study found a wide range of facilitators and barriers to the implementation of our novel PROMise model of care, where patients with axSpA will be monitored remotely through laboratory tests and PROM results. We also presented the ERIC strategies that would possibly mitigate the identified CFIR barriers. These findings will guide the implementation of the PROMise model care in patients with axSpA. Future works should evaluate the implementation success of the improved PROMise model of care.

## Supplementary material

10.2196/82480 Multimedia Appendix 1 Interview guide for patients and health care professionals.

10.2196/82480 Checklist 1 COREQ (Consolidated Criteria for Reporting Qualitative Research): 32-item checklist.

## Abbreviations

axSpA: axial spondyloarthritis
BASDAI: Bath Ankylosing Spondylitis Disease Activity Index
CFIR: Consolidated Framework for Implementation Research
COREQ: Consolidated Criteria for Reporting Qualitative Research
ERIC: Expert Recommendations for Implementing Change
HCP: health care professional
HRQoL: Health-Related Quality of Life
PROM: patient-reported outcome measure
PROMise: PROMs-based model of care
