# Supplementary material for: Facilitators and Barriers to Implementing a Remote Monitoring Model of Care for Stable Patients With Axial Spondyloarthritis Using the Consolidated Framework for Implementation Research: Qualitative Study
Source: J Med Internet Res. 2026 Apr 30;28:e82480. doi: 10.2196/82480 (PMC13131829; doi:10.2196/82480)
Supplement: Multimedia Appendix 1 [file jmir-v28-e82480-s001.docx]

## Interview guide for clinician or allied health or nurses or ancillary staff

## Introduction:

Good morning. The purpose of this discussion/interview is to find out about your views regarding the PROMs-based care in axial Spondyloarthritis in SGH. PROMs are questionnaires asking about how you feel about your health and well-being. PROMs-based care is a new model of care that implements PROMs as part of patient’s care and uses PROMs to help in assessment of patient conditions. The interview will last for approximately 1-2 hours.

Identifiers will not be recorded. Identifiers are personal data that could make you identifiable like name, national registration identity card (NRIC), nationality, passport information, date of birth, and telephone number. The interview will be audiotaped and transcribed for analysis.

## Knowledge & Beliefs about the Intervention

1. What do you know about PROMs-based care and their application to the clinical setting? (Probe: interpretation of PROMs-based care)

## Evidence Strength & Quality

1. What kind of information or evidence are you aware of that shows whether or not the implementation PROMs based care in Spondyloarthritis (SpA) will be successful? (Probe: published literature, colleagues, other organisations)
   - How does this affect your perception about the current project to implement PROMs?

## Self-efficacy

1. How confident are you of implementing PROMs-based care in SGH RHI? What barriers do you think prevent you from using referring patients to PROMs-based care in SGH? Why?
2. How confident do you think your colleagues feel about implementing PROMs-based care in SpA or other disease? Are there any other barriers that they might face? Why?
3. What kind of training and support do you think is required to prepare you and your colleagues for the implementation of PROMs-based care? (Probe: reading materials, people to consult about questions, sharing in meeting)

## Relative Advantage

1. Does the organisation or your department have existing programmes similar to the current project to implement PROMs-based care? If yes:
   - How do you think the programme is going? Why?
   - How does the current programme compare to this project to implement PROMs-based care? (Probe: strengths, weaknesses)
2. Do you know of other alternatives to the current project to implement PROMs-based care? How do the alternatives compare with this project? (Probe: advantages, disadvantages)
3. Do you have other suggestions or ideas that you would rather implement?
   - Can you describe that intervention?
   - What advantages do you think this suggestion/ idea has?
4. How do you feel about the implementation of PROMs-based care in SGH Rheumatology?
   - Do you think it will be effective? Why or why not?
   - Do you have any feelings of anticipation? Stress? Enthusiasm? Why?

## Adaptability

1. What kinds of changes do you think you will make to this project to implement PROMs-based care so that it will work effectively in SGH Rheumatology?
   - Why do you think these changes should be made? How will you know if these changes are appropriate?
   - How do you think these changes should be made?
   - Who will decide whether changes are needed to be made?
2. Are there any components that should not be altered? Why?

## Complexity

1. How complicated is this project to implement PROMs-based care? (Probe: duration, scope, intricacy, number of steps involved, similarities/ differences from previous practices, stakeholder support)

## Patient Needs & Resources

1. What do you think are the current needs of the patients? Why do you say so?
2. How well do you think this project to implement PROMs-based care will meet the needs of the patients in SGH Rheumatology?
   - In what ways do you think their need will be met? (Probe: greater agency over their health, reduced costs)
3. How do you think the patients will respond to this project to implement PROMs-based care?
   - What barriers do you think they will face?
   - What information or evidence should be provided to them to encourage their use? (Probe: ease of access, benefits to health)
4. Do you use PROMs in your clinical practice? If yes, what are your experiences related to PROMs?
   - What are your perceptions of it? What are the advantages and disadvantages of it?
   - Do you think it has been useful in clinical practice? Why or why not?
   - What are patients’ perceptions of it? How receptive have patients been? What feedback have they gave?

## Peer Pressure

1. To what extent would this project to implement PROMs-based care provide an advantage SGH RHI? What advantages would there be?

## Structural Characteristics

1. How will the infrastructure of your organisation or department affect the implementation of the intervention? (Probe: IT system, age, size, physical layout)
2. What kinds of infrastructure changes will be needed to facilitate the implementation of PROMs-based care?
3. Could you describe the process that will be needed to make these changes? (Probe: approval process, stakeholder buy-in, policy changes)

## Culture and implementation climate

1. Could you describe the organisation or department’s culture? (Probe: general beliefs, values, assumptions)
   - Types of culture: team culture, hierarchical, entrepreneurial, market culture
2. How do you think the organisation or department's culture will affect the implementation of PROMs-based care?
   - Can you describe an example that highlights this?
3. How essential are PROMs-based care in meeting the goals of the organisation/ department?
4. Are there any current programmes/ practices/ processes in your organisation/ department that are related to the use of PROMs-based care?
   - What do you think about it? (Probe: Whether it meets the existing needs/ goals)
   - How do you think the current project to implement PROMs-based care will fill current gaps or complement the programme in the organisation/ department
5. How receptive do you think the organisation or department will be to the implementation of PROMs-based care?
   - How well does the implementation of PROMs-based care fit with the current practices within the organisation or department? (Probe: shared decision making vs directives by doctor)
   - If there is a good fit, how can PROMs-based care be integrated into current processes?
   - If there is a poor fit, what are the likely conflicts? What are the solutions to integrate it into current processes?
6. To what extent are new ideas embraced and used to make improvements in the organisation or department?
   - Can you describe a recent example?
7. What kinds of high-priority initiatives or activities are already happening in your organisation or department?
   - Do you think this project to implement PROMs will conflict with or complement these initiatives? (Probe: yourself, colleagues, department, organisation)
   - How does the implementation of PROMs-based care compare with the other high-priority initiatives?

### **Organizational Incentives & Rewards**

1. What kinds of incentives do you think should be in place to ensure the successful implementations of PROMs-based care? (Probe: support from supervisor, rewards)
2. What incentives are currently available that will encourage the implementation of PROMs-based care?

### **Learning Climate**

1. Can you describe a recent implementation of a new initiative or programme?
   - Describe the new initiative /programme and the motivation to improve/implement it?
   - What factors helped make it successful/fail?
   - Who were the key "players" and their roles? How they helped/ hindered?
   - What was your involvement?
   - Were people happy with the outcome/initiative?

### **Leadership Engagement**

1. Who are the key individuals or stakeholders in the organisation or department required to ensure the success of the implementation of PROMs-based care?
   - What kind of support or actions are required from them to make the implementation of PROMs-based care successful?
   - How will their support help in the implementation? (Probe: influence others to use)
   - What are their current perceptions about the implementation of PROMs-based care? How do their attitudes vary?
   - What might be appealing to them? What kind of evidence or supporting information is required to get their support?
   - What types of barriers might they create?

### **Available Resources**

1. What resources do you think will be required for the implementation of PROMs-based care?
   - What difficulties do you foresee in obtaining these resources?

### **Champions**

1. Other than the key stakeholders, are there people in your organization who are likely to champion (go above and beyond what might be expected) the implementation of PROMs-based care?
   - What position do these champions have in your organization?
   - How do you think they will help with implementation?

**Interview guide for patients**

## Introduction:

Good morning. The purpose of this discussion/interview is to find out about your views regarding the implementation of PROMs in your routine clinical care. PROMs are questionnaires asking about how you feel about your health and well-being. PROMs-based care is a new model of care to implement PROMs as part of your care and uses PROMs to help in assessing your condition. The interview will last for approximately 1-2 hours.

Identifiers will not be recorded. Identifiers are personal data that could make you identifiable like name, national registration identity card (NRIC), nationality, passport information, date of birth, and telephone number. The interview will be audiotaped and transcribed for analysis.

## Knowledge & Beliefs about the Intervention

1. What do you know about PROMs-based care and their relevance to your medical condition?

## Self-efficacy

1. How confident are you of participating in PROMs-based care? Why?
   1. How complicated do you think this will be for you?
2. How confident do you think other patients participating in PROMs-based care? Why?
   1. How complicated do you think this will be for other patients?
3. What kind of information or support do you think is required to help you and other patients to participate in PROMs-based care PROMs-based care?
   1. What difficulties do you foresee in obtaining these resources?

## Relative Advantage

1. Do you know of other alternatives to the current plan to integrate PROMs-based care in your routine clinical care? How do the alternatives compare with this project? (Probe: advantages, disadvantages)
2. Do you have other suggestions or ideas that you would rather implement?
   1. Can you describe that intervention?
   2. What advantages do you think this suggestion/ idea has?

## Adaptability

1. What kinds of changes do you think are required to increase patients’ receptiveness towards the PROMs-based care?
   - Why do you think these changes should be made?
   - How do you think these changes should be made?
2. Are there any components that should not be altered? Why?

## Patient Needs & Resources

1. How well do you think PROMs-based care will meet your needs as well as the needs of the patients?
   1. In what ways do you think their need will be met? (Probe: greater agency over their health, reduced costs)
2. How do you feel if PROMs-based care were implemented in your routine clinical consultations?
   1. Do you think it will be effective? Why or why not?
   2. Do you have any feelings of anticipation? Stress? Enthusiasm? Why?
   3. What problems do you think you might face?
   4. What will encourage you to participate in PROMs-based care?
3. How do you think other patients will respond to PROMs-based care?
   1. What barriers do you think they will face?
   2. What information or evidence should be provided to them to encourage their use? (Probe: ease of access, benefits to health)

### **Available Resources**

1. What resources do you think will be required for the implementation of PROMs-based care?
   1. What difficulties do you foresee in obtaining these resources?
