# Supplementary material for: Facilitators and Barriers to Implementing a Remote Monitoring Model of Care for Stable Patients With Axial Spondyloarthritis Using the Consolidated Framework for Implementation Research: Qualitative Study
Source: J Med Internet Res. 2026 Apr 30;28:e82480. doi: 10.2196/82480 (PMC13131829; doi:10.2196/82480)
Supplement: Checklist 1 [file jmir-v28-e82480-s002.pdf]

Supplementary Table 1. Consolidated criteria for reporting qualitative research (COREQ): 32-item checklist

Item number	Guide questions	Reported on
**Domain 1: Research team and reflexivity**		
Personal Characteristics		
1. Interviewer/facilitator	Which author/s conducted the interview or focus group?	In Methods (page 4)
2. Credentials	What were the researcher's credentials? E.g. PhD, MD	PhD, BSc
3. Occupation	What was their occupation at the time of the study?	Staff at a medical research center
4. Gender	Was the researcher male or female?	Females
5. Experience and training	What experience or training did the researcher have?	Training in social science and health services research
Relationship with participants		
6. Relationship established	Was a relationship established prior to study commencement?	No relationship was established prior to study commencement. In Methods (page 4)
7. Participant knowledge of the interviewer	What did the participants know about the researcher? e.g. personal goals, reasons for doing the research	Participants were aware of the primary purpose of the study (page 3)
8. Interviewer characteristics	What characteristics were reported about the inter viewer/facilitator? e.g. Bias, assumptions, reasons and interests in the research topic	Interviewer has experience in conducting qualitative interviews (in data collection). (page 3)
**Domain 2: study design**		
Theoretical framework		
9. Methodological orientation and Theory	What methodological orientation was stated to underpin the study? e.g. grounded theory, discourse analysis, ethnography, phenomenology, content analysis	Deductive (CFIR framework) (page 4)
Participant selection		
10. Sampling	How were participants selected? e.g. purposive, convenience, consecutive, snowball	Purposive sampling was employed (in Methods) (page 5)

11. Method of approach	How were participants approached? e.g. face-to-face, telephone, mail, email	Face-to-face (in Methods) (page 4)
12. Sample size	How many participants were in the study?	32 (in Results) (page 5)
13. Non-participation	How many people refused to participate or dropped out? Reasons?	0
Setting		
14. Setting of data collection	Where was the data collected? e.g. home, clinic, workplace	Remotely (Zoom) (page 3)
15. Presence of non-participants	Was anyone else present besides the participants and researchers?	No
16. Description of sample	What are the important characteristics of the sample? e.g. demographic data, date	Characteristics of the sample were described (in Results page 5; Table 1)
Data collection		
17. Interview guide	Were questions, prompts, guides provided by the authors? Was it pilot tested?	In Methods (page 3)
18. Repeat interviews	Were repeat interviews carried out? If yes, how many?	No
19. Audio/visual recording	Did the research use audio or visual recording to collect the data?	Yes, in Methods (page 4)
20. Field notes	Were field notes made during and/or after the interview or focus group?	Field notes were made. (page 4)
21. Duration	What was the duration of the inter views or focus group?	20 – 60 min (in Methods). (page 4)
22. Data saturation	Was data saturation discussed?	In Results (page 5)
23. Transcripts returned	Were transcripts returned to participants for comment and/or correction?	No
**Domain 3: analysis and findings**		

Data analysis		
24. Number of data coders	How many data coders coded the data?	Four coders (in data analysis). (page 4)
25. Description of the coding tree	Did authors provide a description of the coding tree?	Yes, but not presented in the manuscript.
26. Derivation of themes	Were themes identified in advance or derived from the data?	Themes were identified deductively according to the CFIR framework (in data analysis). (page 5)
27. Software	What software, if applicable, was used to manage the data?	NVivo was used (in data analysis). (page 5)
28. Participant checking	Did participants provide feedback on the findings?	No
Reporting		
29. Quotations presented	Were participant quotations presented to illustrate the themes/findings? Was each quotation identified? e.g. participant number	Quotations were presented and participant number provided (Table 2-3)
30. Data and findings consistent	Was there consistency between the data presented and the findings?	Consistency was checked (in Results). (page 7-12)
31. Clarity of major themes	Were major themes clearly presented in the findings?	Major themes were clearly presented (in Results page 7-12; Table 2-3)
32. Clarity of minor themes	Is there a description of diverse cases or discussion of minor themes?	Yes, in the Results (page 12)
